# TaNAC1 acts as a negative regulator of stripe rust resistance in wheat, enhances susceptibility to *Pseudomonas syringae*, and promotes lateral root development in transgenic *Arabidopsis thaliana*

**DOI:** 10.3389/fpls.2015.00108

**Published:** 2015-02-27

**Authors:** Fengtao Wang, Ruiming Lin, Jing Feng, Wanquan Chen, Dewen Qiu, Shichang Xu

**Affiliations:** State Key Laboratory for Biology of Plant Diseases and Insect Pests, Institute of Plant Protection, Chinese Academy of Agricultural SciencesBeijing, China

**Keywords:** wheat, stripe rust, disease resistance, BSMV-VIGS, jasmonic acid

## Abstract

Plant-specific NAC transcription factors (TFs) constitute a large family and play important roles in regulating plant developmental processes and responses to environmental stresses, but only some of them have been investigated for effects on disease reaction in cereal crops. Virus-induced gene silencing (VIGS) is an effective strategy for rapid functional analysis of genes in plant tissues. In this study, *TaNAC1*, encoding a new member of the NAC1 subgroup, was cloned from bread wheat and characterized. It is a TF localized in the cell nucleus, and contains an activation domain in its C-terminal. *TaNAC1* was strongly expressed in wheat roots and was involved in responses to infection by the obligate pathogen *Puccinia striiformis* f. sp. *tritici* and defense-related hormone treatments such as salicylic acid (SA), methyl jasmonate, and ethylene. Knockdown of *TaNAC1* with barley stripe mosaic virus-induced gene silencing (BSMV-VIGS) enhanced stripe rust resistance. *TaNAC1*-overexpression in *Arabidopsis thaliana* plants gave enhanced susceptibility, attenuated systemic-acquired resistance to *Pseudomonas syringae* DC3000, and promoted lateral root development. Jasmonic acid-signaling pathway genes *PDF1.2* and *ORA59* were constitutively expressed in transgenic plants. *TaNAC1* overexpression suppressed the expression levels of resistance-related genes *PR1* and *PR2* involved in SA signaling and *AtWRKY70*, which functions as a connection node between the JA- and SA-signaling pathways. Collectively, TaNAC1 is a novel NAC member of the NAC1 subgroup, negatively regulates plant disease resistance, and may modulate plant JA- and SA-signaling defense cascades.

## INTRODUCTION

Crop plants provide most of the world’s food intake. A plethora of pathogens including viruses, bacteria, fungi, oomycetes, and nematodes cause severe yield losses in crop production. Fortunately, plants have evolved sophisticated defense mechanisms to cope with biotic stresses (Jones and Dangl, 2006; Howe and Jander, 2008). Multiple defense mechanisms, including basal resistance, PAMP-triggered immunity (PTI) and effector-triggered immunity (ETI), are activated at different stages of pathogen infection. Plant defense systems translate pathogen-induced early signaling events into activation of effective defense responses, most of which depend on the action of plant phytohormones such as salicylic acid (SA), jasmonates (JAs), ethylene (ET), abscisic acid (ABA), auxins, cytokinins (CKs), and gibberellins (GAs; Pieterse et al., 2012). The actions of JAs and SA as signals in the regulation of plant immune responses are well established. The JA-signaling pathway is primarily effective in mediating resistance against necrotrophic pathogens and herbivores, while the SA-signaling pathway primarily functions in mediating resistance to biotrophic pathogens. The JA and SA defense pathways generally antagonize each other; thus, elevated resistance against necrotrophs is often correlated with increased susceptibility to biotrophs (Glazebrook, 2005; Derksen et al., 2013; Vos et al., 2013).

In addition to hormones, plant defense responses require diverse types of transcription factors (TFs) involved in downstream signaling pathways. TF families such as bZIP, bHLH, ERF/AP2, MYB, MYC, NAC, and WRKY play important roles in disease resistance (Singh et al., 2002). Since the first NAC (NAM, ATAF1/2, and CUC1)-type TF NAM (no apical meristem) was identified in *Petunia* (Souer et al., 1996), many other NAC-type genes have been found in different plant species and constitute a large plant-specific TF family. Based on several whole plant genome sequences, genome-wide identifications of NAC-type transcription factors (NAC-TFs) have been carried out. These studies predicted 117 NAC-TFs in *Arabidopsis thaliana* (Ooka et al., 2003), 163 in *Populus trichocarpa* (Hu et al., 2010), 151 in *Glycine max* (Le et al., 2011), 152 in *Solanum tuberosum* (Singh et al., 2013), 147 in *Vitis vinifera* (Wang et al., 2013), 110 in *Malus domestica* (Su et al., 2013), 74 in *Oryza sativa* (Nuruzzaman et al., 2010), 180 in *Setaria italica* (Puranik et al., 2013), and 145 in *Gossypium raimondii* Ulbr (Shang et al., 2013). Typically, most NAC proteins share a highly conserved N-terminal NAC domain, which usually consists of five subdomains (A–E) and a well-diversified C-terminal transcription regulatory (TR) region.

NAC-type transcription factors are involved in diverse plant biological regulation processes, including developmental programs, plant senescence control, secondary cell wall formation, and various biotic and abiotic stress responses (Olsen et al., 2005; Nakashima et al., 2012; Puranik et al., 2012). In addition to being involved in plant responses to biotic stresses some NAC proteins have dual roles as positive or negative regulators of response to different pathogens (Seo et al., 2010). For example, *ATAF1* and its barley homolog *HvNAC6* enhanced resistance to the biotrophic fungus *Blumeria graminis* f. sp. *graminis* (*Bgh*; Chen et al., 2013), but attenuated resistance to pathogens such as *Pseudomonas syringae*, *Botrytis cinerea* and *Alternaria brassicicola* (Wang et al., 2009; Wu et al., 2009). Most NACs that function in biotic stress regulation belong to the SNAC (stress-responsive NAC) subgroup. Some members of the NAC-TF family are multifunctional mediators of both development and stress signaling, and 20–25% of *NAC* genes function in at least one or more stress response(s) (Fang et al., 2008). For example, members of the NAC1 subgroup have roles in both development and stress response. It was clearly shown that *NAC1* (ANAC021/ANAC022) directed lateral root formation in *Arabidopsis* through the auxin signaling pathway whereas *TaNAC7* was induced by dehydration, salinity and cold (Xie et al., 2000, 2002; Tang et al., 2012). However, knowledge of the functions of most members in the NAC1 subgroup in disease response is limited.

Bread wheat (*Triticum aestivum*, genomes AABBDD, 2n = 6X = 42) is a globally important crop. As with other plants, stress factors such as drought, high salinity and disease limit productivity and reduce yield. Several characterized NAC-TFs in the wheat genome function in regulating nutrient transportation, development, disease resistance, and stress tolerance (Xie et al., 1999; Uauy et al., 2006; Xia et al., 2010a,b; Xue et al., 2011; Mao et al., 2012). Recently, the genomic sequences of diploid wild einkorn wheat (*Triticum urartu*, AA, 2n = 2X = 14) and diploid goat grass (*Aegilops tauschii*, DD, 2n = 2X = 14) were sequenced, and 76 and 117 NAC-TFs were predicted in the *T. urartu* and *A. tauschii* genomes, respectively. As bread wheat evolved from natural hybridization between cultivated tetraploid emmer wheat and diploid goat grass, the number of NAC-TFs in bread wheat should be much higher than in the parental diploid species and could show greater genetic diversity. However, the exact roles of most wheat NAC-TFs in response to biotic stresses remain unknown.

Previously we isolated an EST sequence encoding a putative NAC-TF, which was differentially expressed during stripe rust infection. The full length of this EST sequence was isolated in the present study. It belongs to the NAC1 subgroup and was named *TaNAC1*. Its expression patterns in response to exogenous hormone applications were analyzed. The biological functions of *TaNAC1* in wheat and transgenic *Arabidopsis* plants were also investigated. *TaNAC1* was involved in both plant disease resistance and development regulation.

## MATERIALS AND METHODS

### PLANT GROWTH, BIOTIC STRESS, AND CHEMICAL TREATMENTS

Wheat near-isogenic line (NIL) *Yr10^∗^6/Taichung 29* containing the *Yr10* gene is resistant to most races of *P. striiformis* f. sp. *tritici* in China, whereas its backcross parent Taichung 29 is highly susceptible. A full length cDNA library of near-isogenic line (NIL) *Yr10^∗^6/Taichung 29* infected by *P. striiformis* race CYR17 was constructed. *P*. *striiformis* races CYR17 and G22, respectively avirulent and virulent to the resistance gene *Yr10* were used in comparative analyses. Wheat seedlings were grown in 8 cm pots and cultivated under 14/10 h day/night photoperiods at 20°C. Seven-day-old seedlings were inoculated with urediniospores of *P*. *striiformis* as previously described (Xia et al., 2010a), and leaf samples were collected at 0, 8, 12, 24, and 48 h post-inoculation (hpi). To profile the expression patterns of *TaNAC1* responding to exogenous plant hormones, 2-week-old seedlings of *Yr10^∗^6/Taichung 29* were treated with 1 mM SA, 0.1 mM MeJA (both SA and MeJA were dissolved in 0.1% ethanol) and ET released from 0.2 mM ethephon, respectively (Zhang et al., 2004), and leaf samples were collected at 0, 1, 3, 6, 12, 24 h post-treatment (hpt). Intact root, stem, leaf, and spike tissues at flowering were sampled separately for tissue-specific expression analyses of *TaNAC1*. After collection all samples were immediately frozen in liquid nitrogen and then stored at -80°C for RNA isolation.

*Arabidopsis thaliana* accession Col-0 was used for genetic transformation. *Arabidopsis* seeds were grown in 8 cm pots containing a mixture of soil and vermiculite (2:1) in a 16 h light/8 h darkness photoperiod (150 μmol m^-2^s^-1^) and 70% relative humidity at 22°C, four plants per pot.

### *Arabidopsis* TRANSFORMATION AND LATERAL ROOT ANALYSIS

To obtain *35S::TaNAC1* transgenic plants, *TaNAC1* cDNA was cloned into the binary vector pCAMBIA-1301 under the control of the cauliflower mosaic virus (CaMV) 35S promoter and transformed into *Arabidopsis* Col-0 by the floral dip method (Bechtold et al., 2003) using *Agrobacterium tumefaciens* strain GV3101. The gene-specific primer pairs included forward primer: 5′-*GGTACC*CGATCCGACCGAGAAGATG-3′ (*Kpn* I site in italics) and reverse primer: 5′-*TCTAGA*GCCATTCCACTAATCTACTG-3′ (*Xba* I site in italics). Positive transgenic lines were firstly screened on half-strength MS plates (with 3% sucrose and 1.2% agar) containing 30 mg L^-1^ hygromycin and then identified by reverse-transcription PCR. Homozygous T_3_ lines were used for further analyses. Col-0 and *TaNAC1* overexpressing lines were planted on half-strength MS plates under a 16 h light/8 h darkness photoperiod at 22°C in a growth chamber for 10 days. Root lengths were then measured and the numbers of lateral roots per plant were recorded. SPSS 16.0 (SPSS Inc., USA^1^) software was used to determine significant differences by one-way ANOVA, taking *P* < 0.05 level as significant according to Duncan’s multiple range test.

### GENE EXPRESSION ANALYSES

Total RNA from each wheat and *Arabidopsis* sample was extracted using TRIZOL reagent according to the manufacturer’s protocol (Invitrogen, USA). Five micrograms (μg) of total RNA per sample was used to synthesize first-strand cDNA with the TransScript II One-Step gDNA Removal and cDNA Synthesis SuperMix Kit (TransGen Biotech, Beijing). Quantitative RT-PCR was performed using GoTaq® qPCR Master Mix (Promega, Wisconsin) and signals were detected with an ABI7500 Real-Time PCR System (Applied BioSystems, New York). Dissociation curves were generated for each reaction to ensure specific amplification. Every reaction was performed three times. Threshold values (*C*_T_) generated from the ABI PRISM 7500 software tool (Applied Biosystems) were employed to quantify relative gene expression using the comparative threshold (2^-ΔΔ^*^C^*^T^) method (Schmittgen and Livak, 2008). *ADP*-*RF* (*ADP-ribosylation factor*, Ta2291; Paolacci et al., 2009) and *PP2A* (*protein phosphatase 2A subunit A3*; Czechowski et al., 2005) transcripts were used as controls in quantitative reverse transcription–PCR (qRT–PCR) analyses of expression patterns of *TaNAC1* and its regulated genes in transgenic *Arabidopsis* plants. Three independent biological replicates were performed for each experiment. SPSS 16.0 (SPSS Inc., USA^2^) software was used to determine significant differences by one-way ANOVA, taking *P* < 0.05 level as significant according to Duncan’s multiple range test, either between expression levels in the same treatment at different times or between experimental and mock treatments. The sequences of qRT-PCR primers are listed in **Table 1**.

**Table 1:** Primers used in qRT-PCR.

Primer name	Forward sequence	Reverse sequence
TaNAC1	TCGTCTTCTACCAGGGGAGG	AGAACACCCTGCATAGCACC
TaADP-RF	GCTCTCCAACAACATTGCCAAC	GCTTCTGCCTGTCACATACGC
PR1	ATGAATTTTACTGGCTTCTCG	TTAGTATGGCTTCTCGTTCACAT
PR2	CAGATTCCGGTACATCAACG	AGTGGTGGTGTCAGTGGCTA
PDF1.2	AAGTGGGACATGGTCAGGGGTT	ACTTGTGTGCTGGGAAGACATAGTT
ICS1	GGCAGGGAGACTTACG	AGGTCCCGCATACATT
LEC	GTTTCGTCTCTGGGTCATGGA	GCAGCAACTTGTTATTCCTTGGA
VSP2	TCAGTGACCGTTGGAAGTTGTG	GTTCGAACCATTAGGCTTCAATATG
ERF1	CGAGAAGCTCGGGTGGTAGT	GCCGTGCATCCTTTTCC
ORA59	CATACAGAGGAGTGAGGAAACG	AATTGAGTACTGCGAGGCTG
MYC2	TCATACGACGGTTGCCAGAA	AGCAACGTTTACAAGCTTTGATTG
ANAC019	GCATCTCGTCGCTCAG	CTCGACTTCCTCCTCCG
ANAC055	GCGCTGCCTCATAGTC	CGAGGAATCCCCTCAGT
ANAC072	TGGGTGTTGTGTCGAAT	ATCGTAACCACCGTAACT
AtWRKY70	CGTCATCATGGTTCGTCCA	CCACCTCCAAACACCATGAGAT
PP2A	TAACGTGGCCAAAATGATGC	GTTCTCCACAACCGCTTGGT

### SEQUENCE ANALYSES AND PHYLOGENETIC TREE CONSTRUCTION

Gene sequences were analyzed using DNAMAN software (Lynnon Biosoft, USA), BLAST and ORF Finder on the NCBI website. Conserved domains and motifs were predicted using the PROSITE Scan tool^3^. The coding sequence of *TaNAC1* was deduced from its isolated cDNA clone, and the closest homologs in other plants were identified using TBLASTX^4^. Multiple alignments of protein sequences were performed with the program Clustal X version 2.0 program (Larkin et al., 2007). The phylogenetic comparison of isolated full-length TaNAC1 and characterized NAC proteins based on the literature (Ooka et al., 2003; Jamar et al., 2010; Christiansen et al., 2011; D’haeseleer et al., 2011; Tang et al., 2012; Feng et al., 2014) and GenBank^5^ were constructed with the neighbor-joining algorithm using MEGA4 program (Tamura et al., 2007). Bootstrap values were calculated from 1000 bootstrap replicates.

### SUBCELLULAR LOCALIZATION

An expression vector containing a complete encoding region of *TaNAC1* cDNA was constructed to confirm nuclear localization of TaNAC1. *Kpn* I and *Xba* I linkers were added to the encoding region and the stop codon was deleted by PCR amplification. The forward primer 5′-*GGTACC*CGATCCGACCGAGAAGATG-3′ (*Kpn* I site in italics) and reverse primer 5′-*TCTAGA*TGACAAGCCGTTCTCCTG-3′ (*Xba* I site in italics) were used. High-fidelity DNA polymerase HIFI Taq (TransGen Biotech, Beijing) was used, and the PCR product was cloned into *Kpn* I and *Xba* I sites of the binary vector pCaMV35S::GFP to produce fusion vector pCaMV35S::TaNAC1-GFP. The expression plasmid and vector control were transformed into onion epidermal cells by particle bombardment using a Biolistic PDS-1000/He gene gun system (BIO-RAD, Hercules, CA, USA). After 24 h of incubation at 25°C, the fluorescence of DAPI and GFP images of the transformed onion epidermal cells were observed under a confocal microscope (Zeiss LSM 510 Meta Confocal Microscope).

### TRANSCRIPTIONAL ACTIVATION ANALYSES IN YEAST

To investigate the transcriptional regulation domains, the complete ORF (S1, 1/290, primer TF1 5′-TC*GAATTC*ATGAGCTCTA- TTGGCATGAT-3′ and primer TR1 5′-TA*CTGCAG*TCAAAGGG- GTGTCCACATCT-3′, *Eco*R I and *Pst* I sites in italics), one C-terminal truncated (S2: 1/180, primer TF1 and primer TR2 5′-TA*CTGCAG*TCTGCTCTTGTAGAACACCCT-3′, *Pst* I site in italics) and two N-terminal truncated (S3: 107/290, primer TF3 5′-TC*GAATTC*GGCGTTGGTGGGGATGCGCA-3′ and TR1; S4: 178/290, primer TF4 5′-TC*GAATTC*CAGAACAAGCAGCCCAA- GGC1-3′ and TR1, *Eco*R I sites in italics) fragments of *TaNAC1* were fused in-frame with encoding region for the GAL4 DNA-binding domain in expression vector pGBKT7. pGBKT7 was used as a negative control. These different constructs were transformed into yeast strain AH109. The transformants were streaked on SD/Trp- and SD/Trp-/His-/Ade- medium plates. After incubation at 28°C for 3 days, the growth status of the transformants was evaluated. The α-galactosidase filter assay was carried out according to the manufacturer’s instructions.

### FUNCTIONAL ANALYSES OF *TaNAC1* IN RESPONSE TO *P. striiformis* INFECTION USING VIGS

BSMV-induced gene silencing assays were conducted as described (Yuan et al., 2011). Specific sequences of target genes *TaPDS* (with primers pairs, vTaPDSf1, 5′-*AAGGAAGTTTAA*CTGCATAAACG- CTTAAAAG-3′ and vTaPDSr1, 5′-*AACCACCACCACCGT*TCT- CCAGTTATTTGAG-3′, LIC adapters in italics) and *TaNAC1* (with primers pairs, vTaNACf1, 5′-*AAGGAAGTTTAA*TAGCGATCCG- ACCGAGAAGA-3′ and vTaNACr1, 5′-*AACCACCACCACCGT*G- CTTGTGGAGGAGGTAGTCG-3′, LIC adapters in italics) were inserted into pCa-cbLIC. Briefly, 2-week-old wheat seedlings of cultivar Funo were cultured in a growth chamber under a 16/8 h day/night photoperiod at 16 ± 2°C, and watered as needed. The entire second leaf surfaces of two-leaf stage wheat seedlings were inoculated with *Nicotiana benthamiana* leaf sap containing BSMV particles by gently sliding pinched fingers from the leaf base to the tip three times. Control plants were mock-inoculated in the same manner with sterile water. Three independent sets of plants were prepared for each treatment (Mock, BSMV, and BSMV-*TaNAC1*). Inoculated seedlings were placed in a growth chamber in darkness and relative humidity of 60% for 24 h at 25 ± 2°C, and then kept under a 16/8 h day/night photoperiod under the same temperature and humidity conditions. Virus phenotypes were observed and photographed after 9 days. At 14 days post virus inoculation, fresh urediniospores of race G22 were collected and inoculated onto the surfaces of third leaves with a paintbrush. Three independent biological replications were performed for each treatment. Stripe rust phenotypes were recorded and photographed 10–15 dpi. The lengths and widths of 60 random uredinia were measured with ocular micrometers at 15 dpi. SPSS 16.0 (SPSS Inc., USA^6^) software was used to determine the significance of differences in length and width between the BSMV and BSMV-*TaNAC1* samples, taking *P* < 0.05 level as significant according to Student’s *t*-tests.

### *IN PLANTA* DISEASE RESPONSE ASSAYS

Homozygous T_3_ generation seeds of transgenic *Arabidopsis* lines were used for pathogen infection assays. *Pseudomonas syringae* pv. *tomato* DC3000 (*Pst* DC3000) and *Pst* DC3000 (*avrRpt*2) were cultivated on King’s B medium plates with corresponding antibiotics at 30°C for 2 days. Pathogen infection and the determination of *in planta* pathogen population growth were conducted as described (Cao et al., 1997). For the systemic acquired resistance assay, three lower leaves were inoculated with the avirulent *Pst* strain DC3000 (*avrRpt*2; OD_600nm_ = 0.1), and plants infiltrated with 10 mM MgCl_2_ served as mock controls. Infected leaves were removed at 3 days post primary infection and two upper leaves were pressure-infiltrated with the virulent *Pst* strain DC3000 (OD_600nm_ = 0.0001). Colony-forming units were scored 3 days later. Plants inoculated only with *Pst* DC3000 were used as controls. Data were subjected to ANOVA using SPSS 16.0 (SPSS Inc., USA^7^), and the significance of differences between different bacterial populations were assessed by the Student *t-*tests.

## RESULTS

### SEQUENCE ANALYSIS OF PUTATIVE *TaNAC1*

**FIGURE 1:**
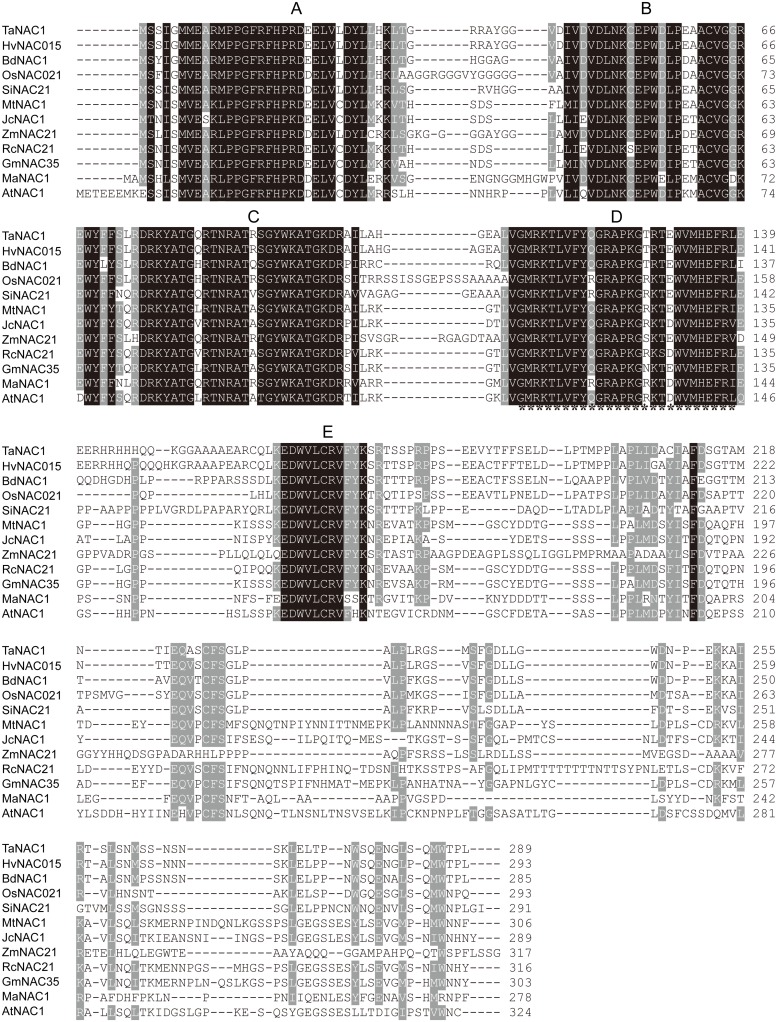
**TaNAC1 sequence analysis.** Amino acid alignment of TaNAC1 with other NAC family members from selected plant species. The numbers on the left indicate amino acid positions. Identical amino acid residues are shaded black representing 100% similarity and dark gray for 75% similarity of conserved amino acids. A putative transcription repression region containing a ‘LVFY’ motif is underlined with asterisks.

Based on the EST sequence of a differentially expressed NAC1 gene during stripe rust infection, the corresponding full length gene was cloned from a cDNA library of wheat near-isogenic line (NIL) *Yr10^∗^6/Taichung 29* by PCR with FastPfu DNA Polymerase. The 1,227 bp cDNA clone contained an 870 bp open reading frame, encoding a putative NAC TF of 289 amino acid residues. Its calculated molecular weight was 32.75 kDa and pI 8.14. Sequence alignment with NAC factors from different plant species showed that this NAC factor contains a conserved NAC DNA-binding domain comprising five subdomains (A–E; **Figure 1**). According to NCBI blastp results the gene was most similar to HvNAC015 with an identity of 92.7%, followed by BdNAC21-22-2 (76.8%), OsNAC21 (73.9%), and AtNAC1. It was named TaNAC1. A NAC transcription repression domain (NARD) containing a conserved hydrophobic ‘LVFY’ motif (Hao et al., 2010) was present in TaNAC1 (**Figure 1**). Accordingly, it may function as a transcription repressor.

**FIGURE 2:**
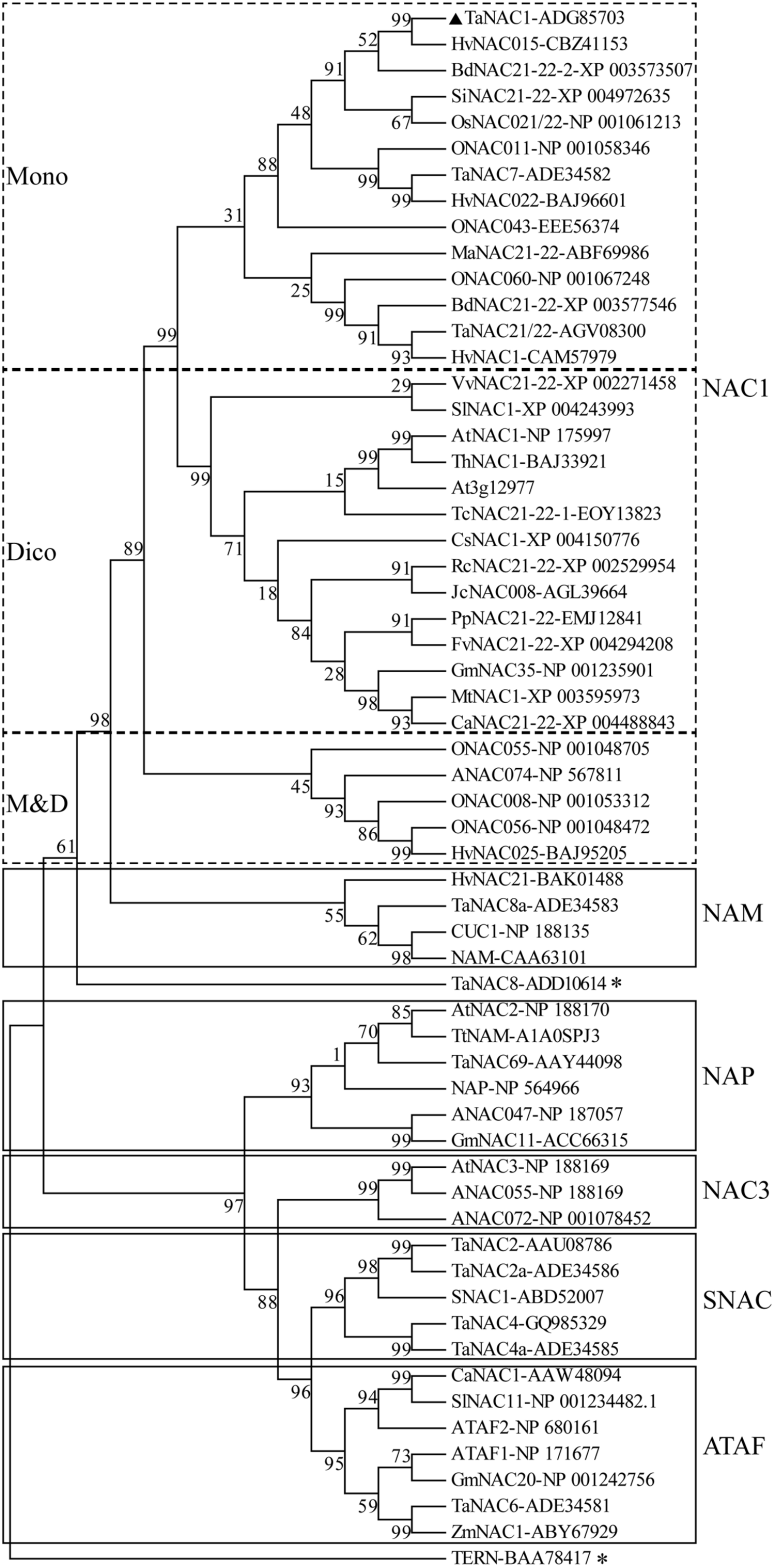
**Phylogenetic tree of NAC transcription factors (NAC TFs).** Phylogenetic tree of all reported NAC TFs from wheat, selected NAC TFs from barley (HvNACs), *Brachypodium distachyon* (BdNACs), *Oryza sativa* (ONACs), *Arabidopsis thaliana* (ANACs), and a few selected NAC factors from other species. TaNAC8 and TERN marked with an asterisk do not belong to any subgroup in this phylogenetic tree. Accession numbers follow the gene names.

A phylogenetic tree was constructed with the putative amino acid sequences of TaNAC1 and NAC family members from other plant species. Phylogenetic analysis revealed that TaNAC1 clustered with the NAC1 subgroup (ANAC021/22) containing 33 members, and those from monocotyledons or dicotyledons clearly clustered into different branches (**Figure 2**).

### SUBCELLULAR LOCALIZATION OF TaNAC1

**FIGURE 3:**
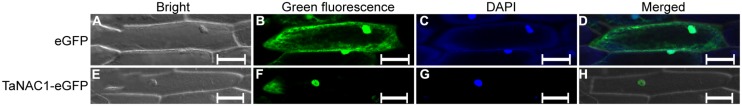
**Subcellular localization of TaNAC1 protein.** *eGFP* vector control and fusion gene *TaNAC1–eGFP* were transiently expressed in onion epidermal cells. The control eGFP protein **(A–D)** and the TaNAC1::eGFP fusion protein **(E–H)** were observed under a confocal microscope. **(A,E)** Bright field images; **(B,F)** Green fluorescent images **(C,G)**. Onion cells were stained with the DNA-binding dye DAPI; **(D,H)** Merged images of bright, green fluorescence, and DAPI signal. eGFP, enhanced the green fluorescent protein; DAPI, 4′, 6-diamidino-2-phenylindole. Bars, 100 μm.

Expression vector pCaMV35S::TaNAC1–GFP was constructed by fusing full-length *TaNAC1* cDNA with the green fluorescent protein (GFP) encoding region, and introduced into onion epidermal cells by particle bombardment in an attempt to determine the subcellular localization of TaNAC1. In the onion cells transformed with GFP vector control, green fluorescence was observed throughout the entire cells (**Figures 3A–D**). In contrast, fluorescence was predominantly detected in the nuclei of cells transformed with the plasmid encoding pCaMV35S::TaNAC1–GFP fusion protein (**Figure 3E–H**), indicating that TaNAC1 is a nuclear-localized protein with roles confines to the cell nucleus.

### TaNAC1 HAS A TRANSCRIPTION ACTIVATION DOMAIN IN ITS C-TERMINAL

**FIGURE 4:**
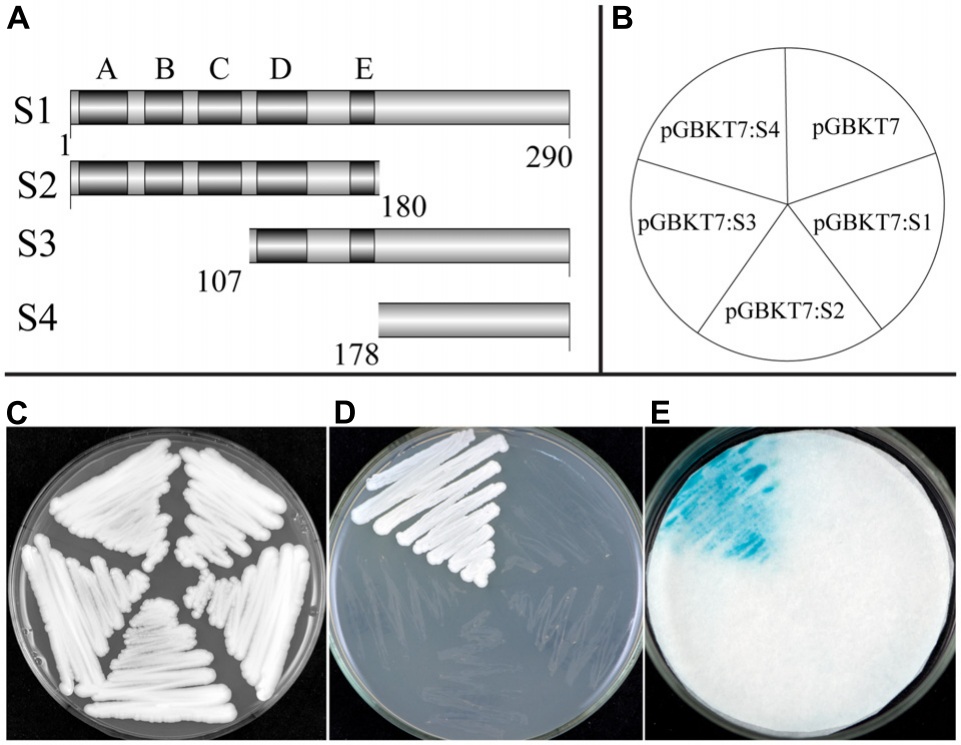
**Transcription activation activity of TF TaNAC1.** Full coding region (S1), N-terminal part (S2), or C-terminal parts (S3 and S4) of *TaNAC1* were fused with the GAL4 DNA-binding domain in vector pGBKT7 **(A)**. Plasmids containing the fusion genes and the empty vector control pGBKT7 were introduced into yeast cells of strain AH109. Yeast transformants of different vectors were plated as shown in the diagram **(B)** and incubated for 2 days at 28°C on an SD/Trp^-^ plate **(C)** and SD/Trp^-^/His^-^/Ade^-^ plate **(D)**. α-galactosidase activities were examined by X-α-Gal staining and kept at 28°C for 8 h **(E)**.

A transcriptional activation assay was investigated using the yeast one-hybrid system. Transformed yeast cells containing the fusion plasmid of full-length cDNA (S1) or the vector control pGBKT7 grew well on selective SD medium without tryptophane (Trp^-^). However, growth was largely inhibited when streaked on SD medium lacking tryptophane, histidine, and adenine (Trp^-^/His^-^/Ade^-^), indicating that TaNAC1 could not function as a transcriptional activator. We made another three fusion constructs containing truncated fragments, S2, the N-terminal containing the NAC DNA-binding domain (amino acid no. 1/180), S3 and S4, the C-terminals (amino acid no. 107/290, 178/290) containing the putative activation domain, with the pGBKT7 vector (**Figure 4A**), and tested transcriptional activation on Trp^-^/His^-^/Ade^-^ medium plates and an α-galactosidase activity assay (**Figure 4B–E**). Only the yeast cells containing fusion construct of S4 could grew fast on selective medium (Trp^-^/His^-^/Ade^-^) showing a positive blue color indicative of strong α-galactosidase activity, and suggesting that this region has a strong transcriptional activation domain. On the contrary, constructs S1 (full length), S2 (N-terminal containing subdomains A–E), and S3 (C-terminal containing subdomains D and E) gave no transactivation activity. We thus confirm that the C-terminal region (amino acid No. 178/290) contained a transcriptional activation domain in TaNAC1.

### ORGAN-SPECIFIC EXPRESSION ANALYSIS OF *TaNAC1*

**FIGURE 5:**
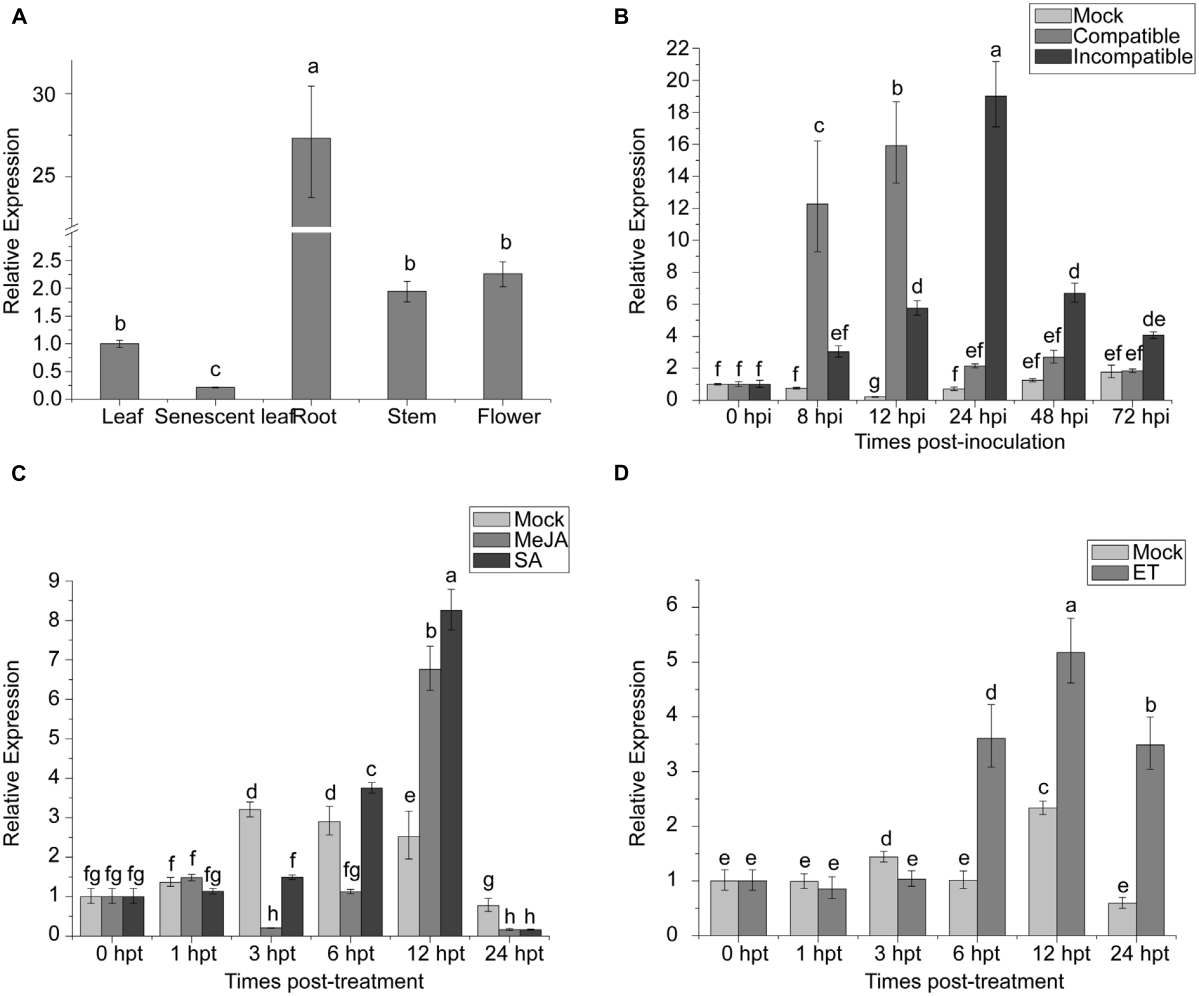
**Expression profiling of *TaNAC1* in wheat. (A)** Relative expression of *TaNAC1* in young or senescent wheat leaves, stems, roots, and flowers; **(B)** Expression patterns of *TaNAC1* in wheat seedlings after inoculation with *Puccinia striiformis* f. sp. *tritici* races *CYR17* and *G22*, which were avirulent and virulent to *Yr10^∗^6/Taichung29*, respectively. The relative expression of *TaNAC1* was calculated from its transcript level in infected seedling leaves compared to that in the mock control across sampling time-points; **(C,D)** Expression patterns of *TaNAC1* in wheat seedling leaves treated with signaling chemicals MeJA, SA, and a mock control of 0.1% (v/v) ethanol, or with ET and its mock control. Results were from three technical replicates. Values are means ± SD (*n* = 3). Similar results were seen in all three biological replicates. Different letter types indicate significant differences by one-way ANOVA, taking *P* < 0.05 level according to Duncan’s multiple range tests of comparisons between different treatment times. MeJA, methyl jasmonate; SA, salicylic acid; ET, ethylene.

NAC proteins can be involved in regulation of plant development. The expression of *TaNAC1* in different wheat tissues was analyzed by qRT-PCR. *TaNAC1* was expressed in all wheat tissues tested, but most strongly in roots, at 27-fold that in young leaves, and least in senescent leaves (**Figure 5A**).

### *TaNAC1* RESPONDS TO BIOTIC STRESS AND HORMONE TREATMENTS

To elucidate whether *TaNAC1* is involved in interaction between wheat and *P. striiformis*, *Yr10^∗^6/Taichung 29* seedlings were inoculated with races CYR17 and G22. The peak expression level of *TaNAC1* appeared earlier and increased faster in the compatible interaction (**Figure 5B**). During the early stages of infection (8–12 hpi) *TaNAC1* expression level in the compatible interaction was significantly higher than in the incompatible interaction, but the inverse relationship occurred in the later stages (24–72 hpi). Expression patterns of *TaNAC1* with exogenous applications of SA, MeJA, ET, and mock treatments were analyzed. For SA treatment compared with the mock control, transcripts of *TaNAC1* initially decreased at 3 hpt, and then increased at 6 hpt to reach a peak level at 12 hpt, before declining sharply at 24 hpt (**Figure 5C**). After MeJA treatment, the expression of *TaNAC1* was decreased at 3 and 6 hpt, then rapidly increased and peaked at 12 hpt (**Figure 5C**). In response to the ET application, expression was slightly induced at 6 hpt, peaked at 12 hpt, and then decreased to a lower level at 24 hpt, although still considerably higher than the mock control at this time point (**Figure 5D**).

### *TaNAC1*-KNOCKDOWN PLANTS SHOW IMPROVED STRIPE RUST RESISTANCE

**FIGURE 6:**
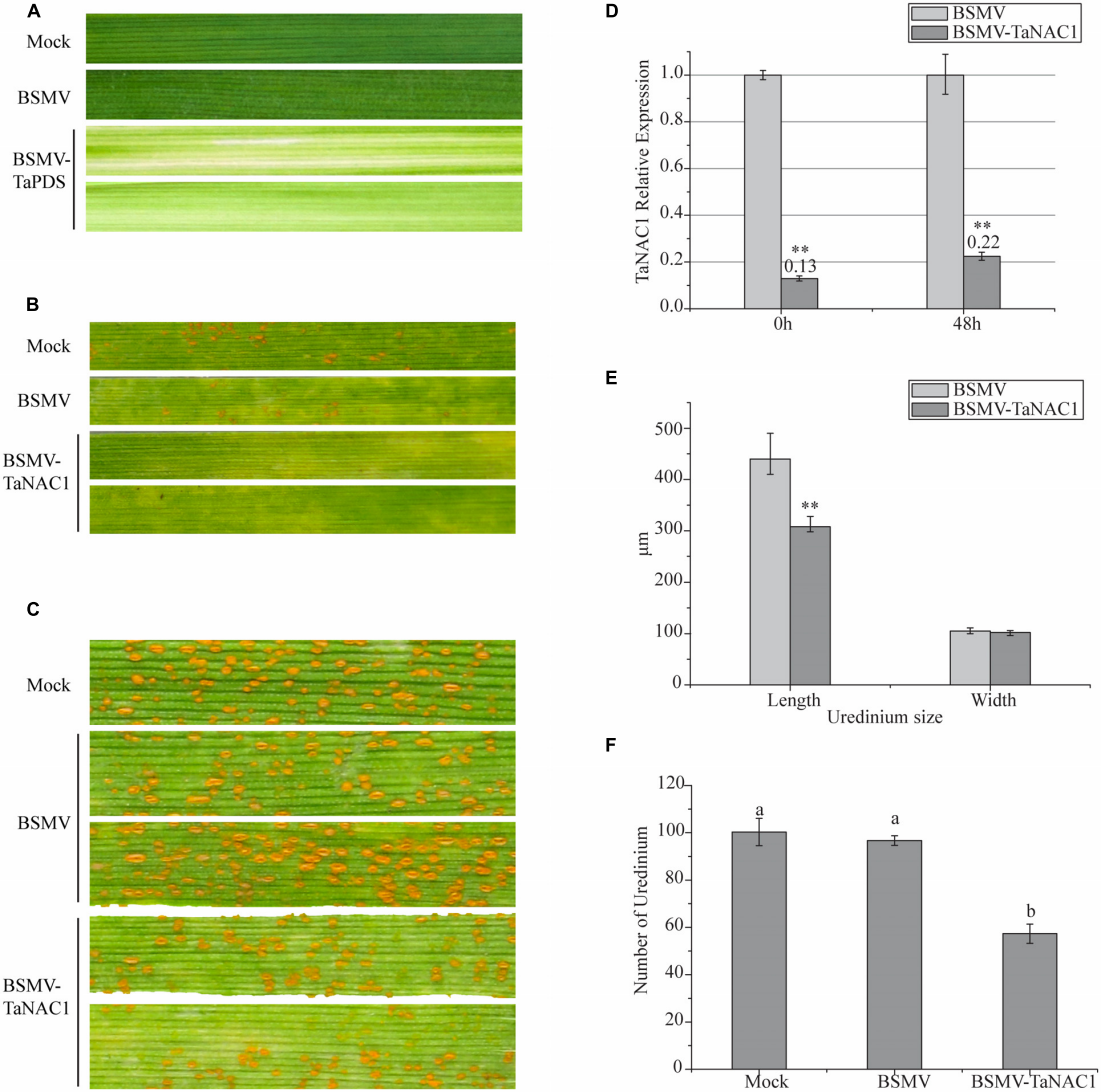
**Functional characterization of *TaNAC1* in response to stripe rust infection using the BSMV-VIGS system. (A)** Phenotypic changes of third seedling leaves of wheat cultivar Funo pre-inoculated with water (Mock), empty BSMV vectors and positive control combination vectors (BSMV-*TaPDS*) at 14 days post-virus treatment. **(B, C)** Phenotypes of third leaves inoculated with *P. striiformis* race G22 were observed at 11 and 15 dpi; the second leaves of these seedlings were pre-inoculated with water (Mock), empty BSMV vectors and BSMV-*TaNAC1*. **(D)** Silence efficiency of *TaNAC1* in *TaNAC1*-knockdown leaves determined by qRT-PCR; third leaves were sampled at 0 and 48 hpi. **(E)** Statistics of 60 uredinia (lengths and widths) on third leaves inoculated at 15 dpi. **(F)** Statistics of uredinia on the 2 cm leaf segments of the third leaves inoculated at 15 dpi. Representative experiment (*n* = 3), each including 60 uredinia and six segments from empty BSMV vectors and BSMV-*TaNAC1*. Error bars indicate SD, asterisks indicate significant differences between BSMV and BSMV-*TaNAC1* samples determined by Student’s *t*-tests (*P* = 0.01) and one-way ANOVA, taking *P* < 0.05 level according to Duncan’s multiple range tests.

In order to determine the role of *TaNAC1* in interaction between the wheat host and the stripe rust pathogen, we constructed a BSMV-*TaNAC1* (barley stripe mosaic virus with a specific fragment of *TaNAC1*) recombinant vector to silence *TaNAC1* expression. The BSMV-inoculated plants displayed mild chlorotic mosaic symptoms at 14 days post-inoculation (dpi), but no obvious defects were observed during further leaf growth. As shown in **Figure 6A**, about 14 days after virus inoculation, typical photobleaching occurred on the leaves of wheat pre-inoculated with BSMV-*TaPDS* (barley stripe mosaic virus with a specific fragment of the wheat phytoene desaturase gene *PDS*) indicating that a genetic interference system applicable to this study was feasible (**Figures 6A,D**). In addition, the virulent *P. striiformis* race G22 successfully infected wheat seedlings previously inoculated with BSMV. Fifteen days after pre-inoculation with the virus, third leaves of these seedlings were inoculated with fresh urediniospores of race G22. At 11 dpi with *P. striiformis* pustules erupted on the leaves of the Mock and viral control plants, but no uredinia appeared on the *TaNAC1*-knockdown seedling leaves (**Figure 6B**). At 15 dpi uredinia were visible (**Figure 6C**). However, there were less uredinial development on *TaNAC1*-knockdown seedling leaves than on the Mock and viral controls (**Figure 6F**). The lengths of *P. striiformis* uredinia on *TaNAC1*-knockdown seedling leaves were significantly shorter than those on the Mock and viral controls at 15 dpi, but there was no difference in uredinial width ((**Figures 6C,E**).

### *TaNAC1* REGULATES ROOT DEVELOPMENT IN TRANSGENIC *Arabidopsis*

**FIGURE 7:**
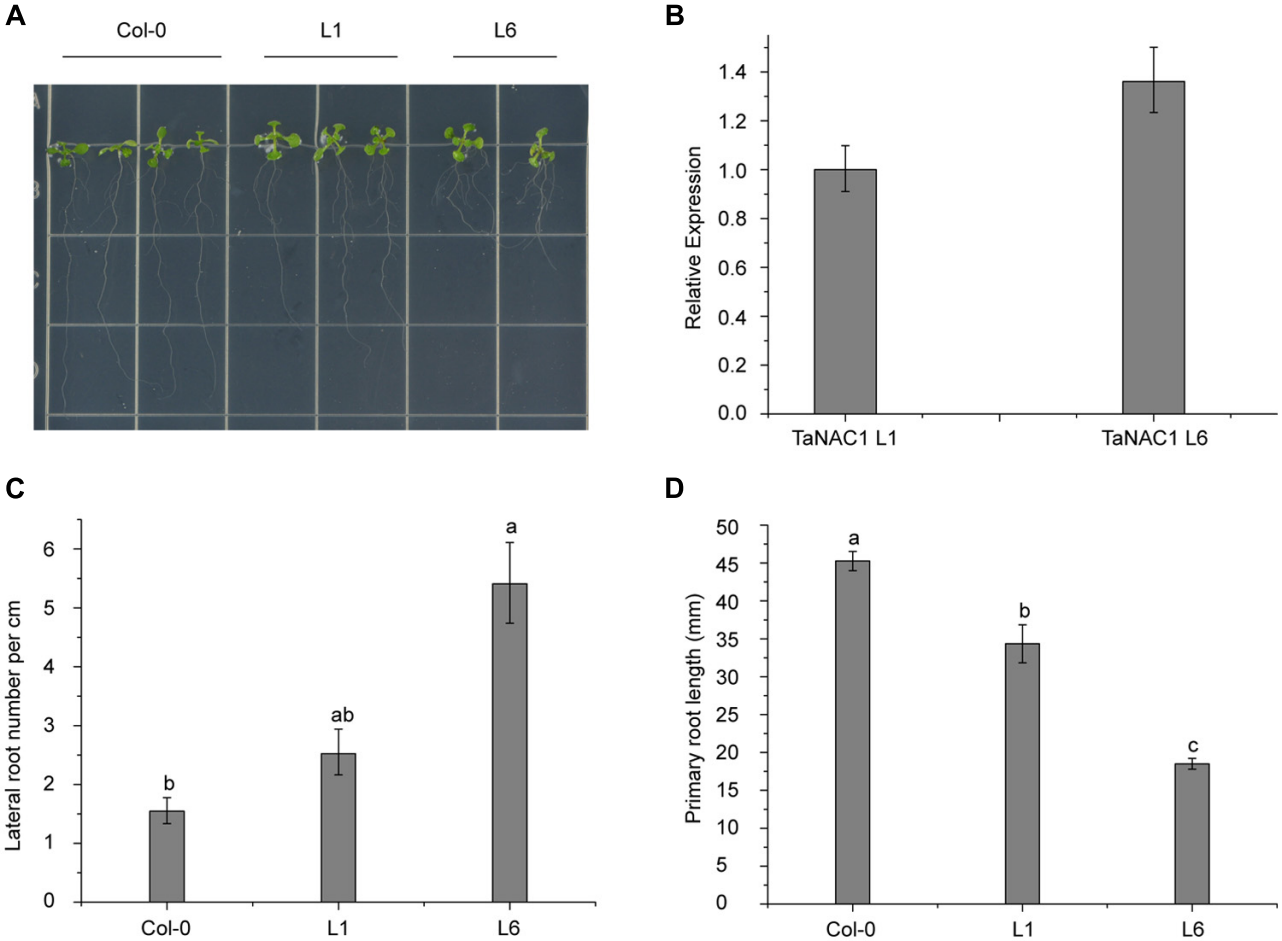
***TaNAC1* regulation of root development on transgenic *Arabidopsis* plants. (A)** *TaNAC1* overexpression promoted 10-day-old transgenic *Arabidopsis* plants of L1 and L6 to produce more lateral roots than the wild type; **(B)** Relative *TaNAC1* expression levels of L1 and L6 analyzed by qRT-PCR; **(C)** Comparison of lateral root density (number of emerged lateral roots per cm of main root) in Col-0 and transgenic lines of L1 and L6; **(D)** Primary root lengths of 10-day-old Col-0 and *TaNAC1*-overexpressing seedlings of L1 and L6. Data show average and SD of 25–30 seedlings and are representative of at least three independent experiments. Different letter types indicate significant differences by one-way ANOVA, taking *P* < 0.05 level according to Duncan’s multiple range tests of comparisons between Col-0 and transgenic lines L1 and L6.

To analyze its biological functions, *TaNAC1* under the control of the CaMV 35S promoter was transformed into *Arabidopsis* Col-0 wild-type plants using *Agrobacterium tumefaciens* strain GV3101. Six positive lines were obtained and two homozygous T_3_ generation transgenic lines (L1 and L6) with different *TaNAC1* expression levels were selected for further biological function analyses. Ten-day-old Col-0 and transgenic seedlings grown vertically on half-strength MS (0.5 × MS) plates were used for observing lateral and primary root development. L6 plants had nearly twice as many lateral roots as L1; and both transgenic lines developed more lateral roots than the wild-type plants (**Figures 7A,C**). However, the primary root lengths of L1 (3.43 cm) and L6 (1.85 cm) plants were 1.10 and 2.65 cm shorter, respectively, than those of wild-type plants (4.52 cm; **Figure 7D**). *TaNAC1* expression level in L6 plants was almost 1.35-fold higher than in L1 plants (**Figure 7B**). Therefore, when *TaNAC1* was overexpressed in *Arabidopsis*, lateral root development in transgenic lines was promoted, but primary root elongation was suppressed. The phenotypes of transgenic *Arabidopsis* root development were closely related to *TaNAC1* expression level.

### *TaNAC1* OVEREXPRESSION ENHANCES SUSCEPTIBILITY OF *Arabidopsis* TO VIRULENT STRAIN *Pst* DC3000

**FIGURE 8:**
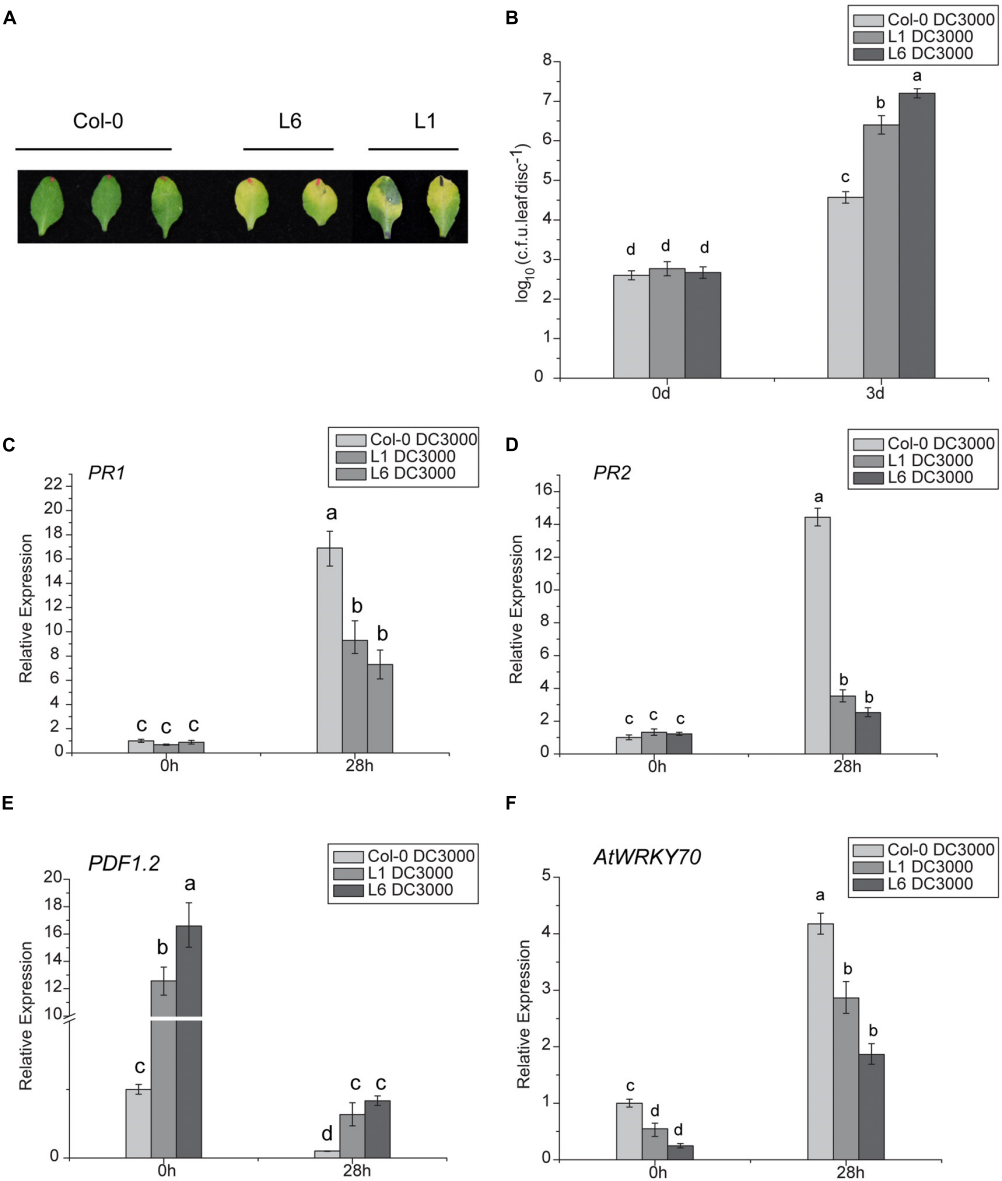
**Analyses of susceptibility of transgenic *Arabidopsis* plants to *Pst* DC3000 and expression patterns of plant defense-related marker genes. (A)** Lesion development on infected leaves of L1, L6, and Col-0 plants 3 days after infiltration with *Pst* DC3000; **(B)** Bacterial populations in infected leaves of wild-type and transgenic lines L1 and L6. Leaves of 4-week-old plants were infiltrated with bacterial suspensions. Nine leaf disks (8 mM diameter) from three different plants were collected 3 days after inoculation. Defense-related marker genes *PR1* **(C)**, *PR2* **(D)**, *PDF1.2* **(E)**, and *AtWRKY70* **(F)** expressions in *TaNAC1*-overexpressing lines L1 and L6 and Col-0 plants at 28 hpi with *Pst* strain DC3000 and in the corresponding controls were analyzed by qRT-PCR. Error bars indicate SD of three technical replicates. Similar results were seen in three biological replicates. Different letter types indicate significant differences by one-way ANOVA, taking *P* < 0.05 level according to Duncan’s multiple range tests.

The DC3000 strain of *Pseudomonas syringae* pv. *tomato* (*Pst* DC3000) was used as a model virulent pathogen for assessing disease susceptibility in transgenic *Arabidopsis* lines. Significant symptom differences developed after infiltrating a low-dose of inoculum (OD_600nm_ = 0.002) of *Pst* DC3000 into leaves of *TaNAC1*-overexpressing transgenic plants L1 and L6, and Col-0. At 48 h post bacterial infiltration larger light yellow lesions were present on infected leaves of L1 and L6 plants compared to slight chlorosis on Col-0, and the disease-associated chlorotic lesions on the leaves of the two transgenics were significantly larger than the control at 3 days post infiltration (**Figure 8A**). Furthermore, transgenic plants supported higher bacterial growth rates than the wild-type plants (**Figure 8B**).

### *TaNAC1* AFFECTS THE EXPRESSION OF DEFENSE-RELATED MARKER GENES

To determine whether enhanced pathogen susceptibility of *TaNAC1* transgenic plants was linked to changes in defense-related gene expression and to gain further insight into possible regulation pathways, the expression patterns of several well-known defense-related marker genes in *TaNAC1*-overexpressing and Col-0 plants infiltrated with *Pst* DC3000 were compared. There was no significant difference in basal expression levels of SA-responsive genes *PR1* and *PR2* between L1, L6 and Col-0 plants. However, both *PR1* and *PR2* in *TaNAC1* transgenic plants and Col-0 greatly increased after infiltration with *Pst* DC3000, and Col-0 plants had a higher expression level than L1 and L6 plants (**Figures 8C,D**). ET- and JA-regulated defense-related marker gene *PDF1*.*2* significantly increased in transgenic plants and the basal transcript levels in L1 and L6 plants were nearly 13-fold and 16-fold higher than that in Col-0 plants (**Figure 8E**). *PDF1.2* expression was suppressed at 28 hpi, but was still significantly higher in L1 and L6 plants than in the wild-type control.

The AtWRKY70 factor functions as a node between the JA- and SA-signaling pathways; expression of *AtWRKY70* is repressed by JA, but activated by SA, and AtWRKY70 acts as a negative regulator of expression of JA-responsive downstream genes and as a positive regulator of SA-induced genes such as *PR1* (Li et al., 2006). *AtWRKY70* expression levels in the present study were lower in *TaNAC1* transgenic lines than Col-0 plants before and after *Pst* DC3000 infection, although its transcription could be induced by *Pst* DC3000 infection. Its expression was apparently suppressed in L1 and L6 plants compared to Col-0 plants (**Figure 8F**). As a consequence, overexpression of *TaNAC1* resulted in significantly higher levels of expression of the ET- and JA-regulated marker gene *PDF1.2*.

### *TaNAC1* INVOLVES ET- AND JA-SIGNALING PATHWAYS

**FIGURE 9:**
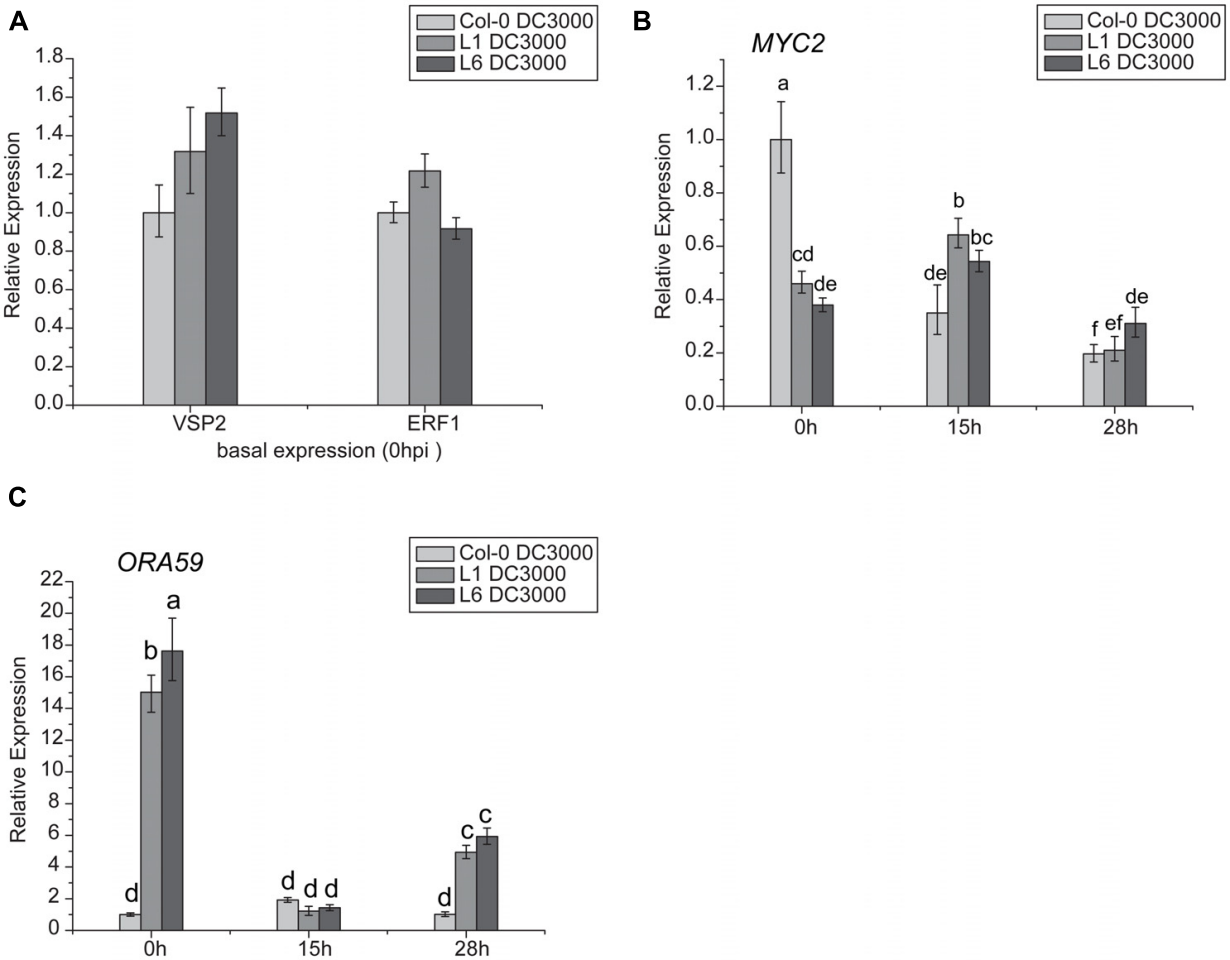
**Jasmonates (JA)-signaling pathways genes showed different expression patterns between *TaNAC1* transgenic and Col-0 plants. (A)** *VSP2* and *ERF1* expressions in uninoculated Col-0 and *TaNAC1-*overexpressing lines L1 and L6; **(B)** *MYC2* and **(C)** *ORA59* expressions in Col-0 and *TaNAC1-*overexpressing L1 and L6 plants inoculated with *Pst* strain DC3000 (OD_600nm_ = 0.001) at 0, 15, and 28 hpi. Error bars indicate SD of three technical replicates. Similar results were seen in three biological replicates. Different letter types indicate significant differences by one-way ANOVA, taking *P* < 0.05 level according to Duncan’s multiple range tests.

Jasmonates signaling occurs via two different branches, and is regulated by *MYC2* or *ERF1*/*ORA59*. *MYC2* is also involved in the ABA- and JA-signaling pathways. In this study, *VSP2* was used as a marker gene for the *MYC2* pathway, and *PDF1.2* for the *ERF1*/*ORA59* pathway. Results indicated that there was no significant difference in *VSP*2 and *ERF1* transcription levels between L1, L6, and Col-0 plants (**Figure 9A**), but basal transcript levels of *ORA59* were significantly higher in L1 and L6 plants than Col-0 at 0 h (**Figure 9C**). Moreover, *MYC2* expression was highly suppressed in *TaNAC1* transgenic plants compared with Col-0 plants before infection (**Figure 9B**).

Because *ORA59* and *MYC2* expression levels were significantly changed in transgenic plants, the expression patterns of these two genes were further analyzed following infection with *Pst* DC3000. The expression level of *ORA59* in Col-0 plants, remained similar after infiltration whereas in L1 and L6 plants it was significantly reduced at 15 hpi and then partially recovered, but was still much higher than in Col-0 at 28 hpi (**Figure 9C**). The *MYC2* transcript level in Col-0 decreased significantly during incubation after infiltration, but in L1 and L6 plants it was slightly increased at 15 hpi and then decreased at 28 hpi (**Figure 9B**), suggesting that JA- and ET-signaling pathways were affected by *TaNAC1* overexpression.

### *TaNAC1* OVEREXPRESSION ATTENUATED SYSTEMIC-ACQUIRED RESISTANCE

**FIGURE 10:**
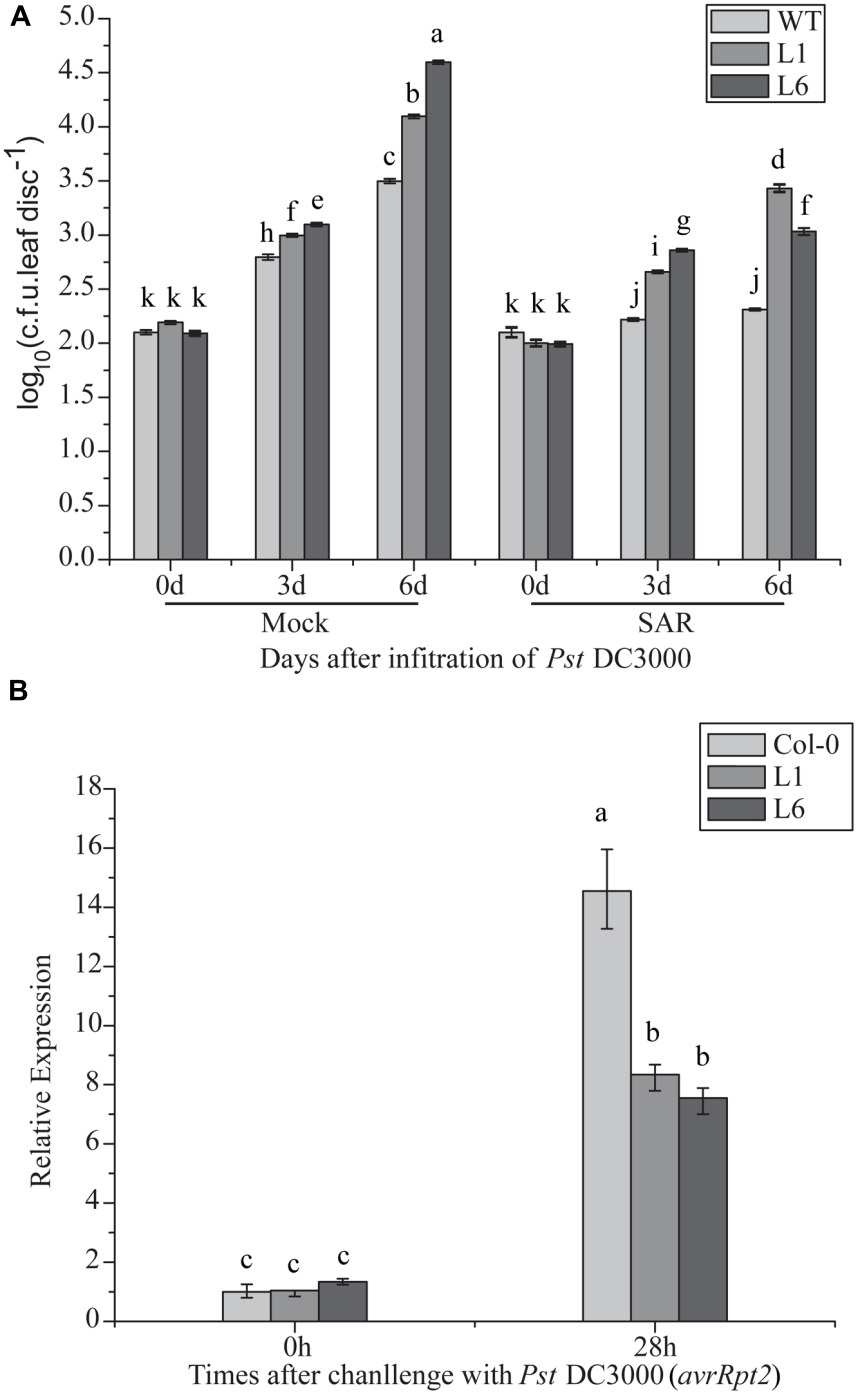
**Induction of systemic acquired resistance (SAR) in *TaNAC1* overexpressing plants. (A)** Three lower leaves on each plant were inoculated with *Pst* strain DC3000 (*avrRpt2*; OD_600nm_ = 0.1; SAR) and plants infiltrated with 10 mM MgCl_2_ served as mock controls. Three days later, two upper uninfected leaves were pressure-infiltrated with virulent *Pst* strain DC3000 (OD_600nm_ = 0.0001). Plants inoculated only with *Pst* DC3000 served as controls for SAR analysis. The *in planta* bacterial titers were determined immediately and 3, 6 days after challenge inoculation. Data represent the mean of 4–10 independent samples with SD. Different letter types indicate significant differences by one-way ANOVA, taking *P* < 0.05 level according to Duncan’s multiple range test of comparisons between populations from different plants or different times. **(B)** *PR1* expression in uninoculated Col-0 and *TaNAC1-*overexpressing lines L1 and L6. Three lower leaves on each plant were inoculated with *Pst* DC3000 (*avrRpt2*; OD_600nm_ = 0.1; SAR). 28 h later, total RNA was extracted from upper non-infected leaves and analyzed for gene expression using qRT-PCR. Error bars indicate SD of three technical replicates. Similar results were observed in three biological replicates. Different letter types indicate significant differences by one-way ANOVA, taking *P* < 0.05 level according to Duncan’s multiple range tests.

Compromised disease resistance in *TaNAC1-*overexpressing plants may affect SAR establishment. To test this hypothesis, changes in the bacterial population with or without SAR treatment were analyzed. Transgenic *TaNAC1* plants supported larger DC 3000 bacterial populations than the control (**Figure 10A**). *PR1* expression level was significantly increased in the transgenic lines after SAR treatment, indicating that *TaNAC1* overexpression did not block SAR establishment, but the *TaNAC1* transgenic plants had a lower *PR1* transcription level than Col-0 when pre-inoculated with an avirulent strain of *Pst* DC3000 (*avrRpt2*; **Figure 10B**). Therefore, SAR treatment induced disease resistance in both *TaNAC1*-overexpressing lines and Col-0 plants compared with the corresponding mock controls. *TaNAC1* overexpression in *Arabidopsis* did not block SAR but compromised its induction of resistance.

## DISCUSSION

NAC-type transcription factors form one of the largest plant-specific TF families, regulating plant development and responses to environmental stimuli (Nakashima et al., 2012; Puranik et al., 2012). Several NAC-TFs have been characterized in wheat (Xie et al., 1999; Uauy et al., 2006; Xue et al., 2006, 2011; Xia et al., 2010a,b; Mao et al., 2012, 2014; Tang et al., 2012; Avni et al., 2014; Feng et al., 2014). For example, TaNAC21/22, a member of the NAC1 subgroup, takes part in regulation of resistance to stripe rust (Feng et al., 2014). The first characterized member of NAC1 subgroup, NAC1, was identified in *Arabidopsis* and primarily regulates plant root development (Xie et al., 2000).

### THE C-TERMINAL OF TaNAC1 HAS TRANSCRIPTIONAL ACTIVATION ABILITY

In this study, a novel member to the NAC-TF family was isolated from bread wheat. Basedonn a neighbor-joining cluster tree of known NAC proteins from *Arabidopsis*, wheat, rice, barley and other species, this NAC factor was classified into the NAC1 subgroup together with NAC1 and At3g12977.1 from *Arabidopsis* and TaNAC21/22 and TaNAC7 from wheat.

We performed transactivation analyses of TaNAC1 in yeast, and the results showed that the full length and N-terminal region of TaNAC1 lacked transcriptional activation ability. But the C-terminal possessed strong transactivation capacity. The N terminus of TaNAC1 was predicted to contain a NARD domain (**Figure 1**), which was recently reported to be a conserved and active repression domain in NAC proteins (Hao et al., 2010). GmNAC20 and TaNAC7, which both belong to the NAC1 subgroup (**Figure 2**), did not have transcriptional activation ability (Tang et al., 2012). So, the NARD domain may remarkably attenuate the transcriptional activation of TaNAC1 factor.

### *TaNAC1* IS INVOLVED IN RESPONSE TO FUNGAL AND BACTERIAL PATHOGENS

Phytohormones such as SA, MeJA, and ET are believed to play key roles in signaling pathways involved in plant responses biotic and abiotic stresses. Each of these molecules, especially SA, transiently induced *TaNAC1* expression (**Figure 5**). Until now, more than 10 wheat NAC-TFs have been reported, but only *TaNAC4* and *TaNAC8*, belonging to the SNAC and OsNAC8 subgroups, respectively, were reported to be induced by MeJA, ET, and pathogen infection. However, SA did not affect the expression of those two genes (Xia et al., 2010a,b).

NAC-type transcription factors are proved to be involved in responses to pathogen attack. For example, *ATAF1-*overexpressing plants increased susceptibility to *Pst* DC3000 and the necrotrophic pathogen *Botrytis cinerea*. *HvNAC6* (Chen et al., 2013), *ANAC019*, *ANAC055*, and *ANAC072* (Zheng et al., 2012), *ATAF2* (Delessert et al., 2005), *OsNAC4* (Kaneda et al., 2009), and membrane-tethered NAC gene *NTL6* (Seo et al., 2010) were also involved in plant–pathogen interactions. Generally, three phases of plant defense are defined, the first pre-invasive defense barrier of the plant (Phase I), the second barrier of early post-invasive defense (Phase II), and transcriptomic and metabolomic re-programming, resulting from a hypersensitive response (Phase III). Phase III is associated with production of intra- and intercellular signals, including defense hormones (Ton et al., 2009). In the present research, *TaNAC1* expression was closely related to the wheat defense response to *P. striiformis*, and was significantly upregulated at an early stage of infection (Phases I and II) in the compatible interaction. The expression levels of *TaNAC1* were partially synchronic with pathogenesis phases described previously (**Figure 5B**). Furthermore, when *TaNAC1* was knocked down, uredinia produced by a virulent *P. striiformis* race appeared later and were smaller on knockdown plants. Therefore, suppression of *TaNAC1* transcription apparently delayed the infection process and partially increased resistance to the virulent *P. striiformis* race.

*TaNAC1* overexpression in *Arabidopsis* enhanced susceptibility of transgenic plants to the virulent *Pst* strain DC3000, promoting population growth of the bacterial pathogen and accelerating the pathogenesis process. But the SAR of *TaNAC1* transgenic plants remained almost intact. These results strongly confirmed that *TaNAC1* functioned as a negative regulator in the basal disease resistance pathway to *Pst* strain DC3000. In *Arabidopsis*, several genes were characterized as negative disease resistance regulators to pathogenic bacteria, including NAC TFs (Kim et al., 2012), *ERF1* (Berrocal-Lobo et al., 2002), WRKY factors (Journot-Catalino et al., 2006; Kim et al., 2006, 2008; Zheng et al., 2007), kinase and kinase phosphatase *MKP* (Anderson et al., 2011), *CBRLK1* (Kim et al., 2009), and *MPK4* (Colcombet et al., 2013).

### *TaNAC1* MAY SUPPRESS RESISTANCE-RELATED GENE EXPRESSION IN SA SIGNALING

Cross-talk between JA, ET, and SA signaling is thought to operate as a mechanism to fine-tune induced defenses activated in response to multiple biotic attackers. SA appears primarily for signaling in plant resistance to biotrophic and hemibiotrophic pathogens such as *P. syringae*. JA plays a role in plant defense against insects and necrotrophic pathogens and may suppress some defense responses through antagonism with SA. *Arabidopsis* plants defective in SA biosynthesis or its signaling were more susceptible to hemibiotrophic bacterial pathogens than wild-type plants, whereas plants with enhanced JA biosynthesis or signaling also support bacterial pathogen population growth. SA levels are usually tightly regulated in dicotyledonous plants. When infiltrated with the virulent *Pst* strain DC3000, SA-regulated *PR* gene expression level decreased in *TaNAC1*-overexpressing plants. Furthermore, infection in the lower leaves of *TaNAC1* transgenic plants by an avirulent *P. syringae* strain induced *PR1* gene expression in upper uninoculated leaves and increased *Pst* DC3000 population growth after inoculation of transgenic plants relative to Col-0 plants. These results strongly proved that TaNAC1 is a negative regulator of *P. syringae*-induced *PR* gene expression in *Arabidopsis*.

ORA59 acts as an integrator of JA- and ET-signaling pathways and is the key regulator of JA- and ET-responsive gene *PDF1.2* (Pre et al., 2008). A recent study showed that the SA pathway inhibits the SCF(COI1)-JAZ complex of JA downstream signaling via a negative effect on the transcriptional activator *ORA59* but without influence on ERF1 expression (Van der Does et al., 2013). When higher levels of *ORA59* transcripts accumulated in plants infiltrated with *Pst* DC3000, additional free SA was needed to reduce *ORA59*, thus priming the SA-signaling resistance pathway. In the present study, ET- and JA-regulated defense-related marker genes *PDF1*.*2* and *ORA59* were expressed at significantly higher levels in transgenic plants than in wild-type, and there was no significant difference in *ERF1* transcription levels. In addition, AtWRKY70 functions as a connection node between JA- and SA-signaling pathways, and is activated by SA but suppressed by JA. This gene was greatly suppressed in the *TaNAC1* overexpression lines, and hence may lead to lower expression levels of SA-regulated genes in *TaNAC1*-overexpressing plants.

In *Arabidopsis*, two major branches of the JA signaling pathway are recognized. One is the MYC branch with marker gene *VSP2*, and another is the ERF branch requires both JA and ET signaling with the marker gene *PDF1.2*. In general, the ERF branch is associated with enhanced resistance to necrotrophic pathogens (Berrocal-Lobo et al., 2002; Lorenzo et al., 2003), whereas the MYC branch is associated with the wound response and defense against insect herbivores (Lorenzo et al., 2004; Kazan and Manners, 2008). In wheat under treatment with MeJA, *TaNAC1* also showed a rapid increase and peaked at 12 hpt. However, the roles of JA in biotrophic and necrotrophic resistances are unclear in many monocot pathosystems. There is no dichotomy between the effectiveness of the JA pathway and the lifestyle of the invading pathogen in rice (De Vleesschauwer et al., 2014), and some reports implicate roles of JA in resistance against (hemi)biotrophic pathogens *Xanthomonas oryzae* and *Magnaporthe oryzae* (Mei et al., 2006; Yamada et al., 2012; Taniguchi et al., 2014), necrotrophic rice pathogens (Taheri and Tarighi, 2010; Peng et al., 2012) and insect herbivores (Zhou et al., 2009). Thus *TaNAC1* may play a positive role in resistance to the necrotrophic pathogens both in *Arabidopsis* and wheat.

Therefore enhanced susceptibility of *TaNAC1*-overexpressing plants to *Pst* DC3000 may result from suppression of resistance-related gene expression in SA signaling by regulation of the JA-signaling pathway.

### *TaNAC1* IS AN IMPORTANT REGULATOR OF ROOT DEVELOPMENT

Several members of the NAC1 subgroup are involved in regulation of plant root development. *Arabidopsis* NAC1 functioned in auxin-mediated signaling and increased the number of lateral roots in transgenic overexpressing plants (Xie et al., 2000; Hao et al., 2011). GmNAC11 and GmNAC20 were reported to be the members of the NAC1 subgroup and increased lateral root number (Hao et al., 2011). In this study, however, GmNAC11 and GmNAC20 clustered with the NAC3 and ATAF1 subgroups, respectively. For MtNAC1, another factor of the NAC1 subgroup, no changes in lateral root development or nodulation occurred on transgenic plants (D’haeseleer et al., 2011). As a novel member of NAC1 subgroup, TaNAC1 has high similarity to its homolog NAC1 of *Arabidopsis*, and its organ-specific expression pattern highly resembles that of *NAC1* (Xie et al., 2000) in being more abundantly expressed in roots than in other organs. Moreover, *TaNAC1*-overexpressing *Arabidopsis* plants developed more lateral roots but primary root elongation was considerably suppressed.

Lateral root development in higher plants is influenced by a wide range of environmental cues, but auxin plays a dominant role (Fukaki et al., 2007; Petricka et al., 2012). JA is also involved in this process by activating auxin biosynthesis and has effects on auxin transport into the root basal meristem (Sun et al., 2009). Application of exogenous JA inhibited plant root growth (Staswick et al., 1992). *Arabidopsis* mutant *JA-induced defective lateral root1* (*jdl1/asa1-1*) repressed lateral root formation, while *anthranilate synthase* α*1* (*ASA1*) was needed for JA-induced auxin biosynthesis. Combined with positive regulation roles in the JA- and ET-signaling pathways *TaNAC1* functions in regulating root development may depend on the JA-signaling pathway.

In brief, *TaNAC1* encodes a new NAC-type TF. Based on the results of the present study, we concluded that *TaNAC1* might negatively regulate wheat defense against *P. striiformis* and *Arabidopsis* response to *Pseudomonas syringae*, as well as being involved in regulation of plant root development.

## Conflict of Interest Statement

The authors declare that the research was conducted in the absence of any commercial or financial relationships that could be construed as a potential conflict of interest.

## Footnotes

1 http://spss.en.softonic.com/

2 http://spss.en.softonic.com/

3 http://prosite.expasy.org/scanprosite/

4 http://www.ncbi.nlm.nih.gov/BLAST/

5 http://www.ncbi.nlm.nih.gov/

6 http://spss.en.softonic.com/

7 http://spss.en.softonic.com/
